# A Patient With Eosinophilic Gastroenteritis Presenting With Severe Abdominal Pain

**DOI:** 10.1002/ccr3.9667

**Published:** 2024-12-06

**Authors:** Sumona Islam, Dewan Saifuddin Ahmed, Nabila Tasneem Khan, Farjana Sultana Rakhi

**Affiliations:** ^1^ Department of Gastroenterology Bangabandhu Sheikh Mujib Medical University Dhaka Bangladesh; ^2^ Delta Hospital Dhaka Bangladesh

**Keywords:** eosinophilia, eosinophilic gastroenteritis, eosinophilic gastrointestinal disorders, eosinophilic infiltration

## Abstract

Eosinophilic gastroenteritis is characterized by eosinophilic infiltration of the stomach and intestine. It is a rare disorder with bizarre presentations, making it difficult to diagnose and often leading to misdiagnoses. It can present with abdominal pain, diarrhea, vomiting, obstruction, ascites, etc. Here, we report a case of a middle‐aged female who presented with severe abdominal pain and was initially suspected to have acute pancreatitis. She received conservative management, but symptoms did not improve. This led to further evaluation, which revealed peripheral blood eosinophilia. Although the mucosa appeared normal on esophagogastroduodenoscopy, biopsy from multiple sites revealed eosinophilic infiltration in the gastric and duodenal lamina propria. The patient was then successfully treated with oral steroid. Due to its varied presentations, eosinophilic gastroenteritis should be considered as the differential diagnosis in different abdominal presentations.

**Summary**

- Eosinophilic gastroenteritis presents with varied symptoms, often mimicking other conditions. High suspicion is necessary for diagnosis, which relies on endoscopic biopsy.
- Early recognition and steroid treatment can lead to rapid symptom resolution and prevent unnecessary suffering.

## 1 Introduction

Eosinophilic gastrointestinal disorders (EGID) are a spectrum of conditions involving different parts of the gastrointestinal tract, principally arising from infiltration of eosinophils in different layers, giving rise to a wide variety of presentations [1, 2]. It is a rare disorder. The true incidence rate is difficult to establish; however, an estimate of 1–30/100,000 is reported [3]. Eosinophilic gastroenteritis (EGE) falls under the EGID spectrum, involving the stomach and the intestines. It can be divided into three types based on the layers involved—mucosa, muscularis, and serosa [4]. Data regarding EGE is scarce, and there is no published case report in Bangladesh to our knowledge. Here, we report a case of a middle‐aged female with severe abdominal pain for one and a half months and later diagnosed as EGE.

## 2 Case Presentation

A 38‐year‐old normotensive female with newly diagnosed diabetes presented with severe abdominal pain for one and a half months. The pain was sudden in onset, severe, diffuse, colicky, aggravated after meal, partially relieved by intravenous analgesics, and not relieved by change in posture. Pain was associated with vomiting, 3–4 times per day, which partially relieved the pain. There was no associated transient visible peristalsis. She had lost 2 kg of weight. Her bowel habit was normal. She denied any history of fever, cough, hemoptysis, contact with smear positive pulmonary tuberculosis patient, swelling in different parts of the body, itching, or night sweat.

With these complaints, she was hospitalized and clinically labeled as a case of acute pancreatitis in a local hospital based on the severity of the pain. Normal serum levels of amylase and lipase were observed, and due to the absence of a definitive diagnosis, conservative management was implemented with analgesics. Initially, pain and vomiting were controlled to some degree, but they reappeared again and became difficult to control.

She had no history of asthma or any atopic disease or known food allergy. She had appendectomy 20 years back and caesarian section 12 years back. She has two children. Her daughter has asthma. There was no family history of gastrointestinal malignancy.

Examination revealed a body mass index (BMI) of 23 kg/m^2^ with no anemia, jaundice, lymphadenopathy, or edema. Abdominal examination revealed no organomegaly or ascites.

## 3 Investigations and Treatment

Complete blood count showed a high while blood cell (WBC) count with > 50% eosinophils. Peripheral blood film revealed mature WBCs. C reactive protein (CRP) was found to be uncharacteristically high. Serum immunoglobulin E (IgE) was increased. Hepatic and renal functions were normal. Breakpoint Cluster Region‐Abelson (BCR‐ABL) was negative. Abdominal and trans‐vaginal ultrasound revealed dilated bowel loops and mild pelvic collection.

**FIGURE 1:**
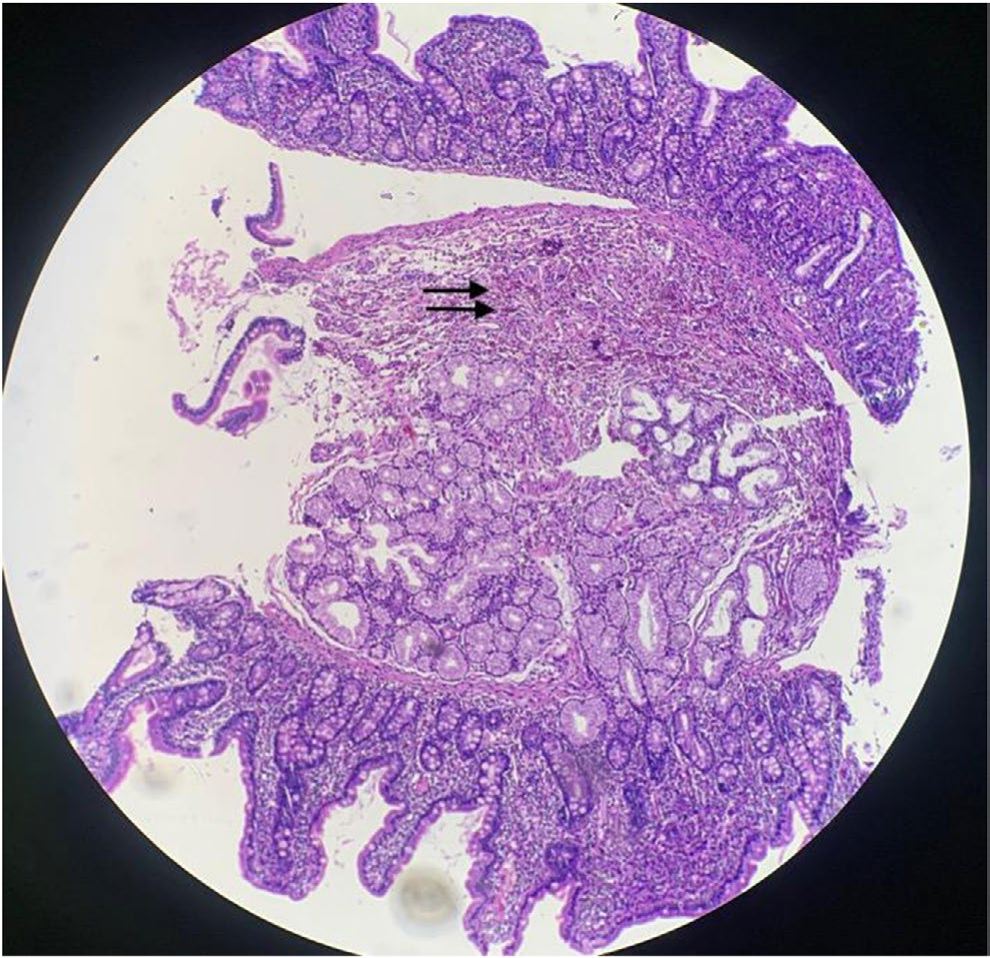
Histopathology from duodenal tissue (4× magnification) showing lamina propria being infiltrated by numerous chronic inflammatory cells, including a fair number of eosinophils.

**FIGURE 2:**
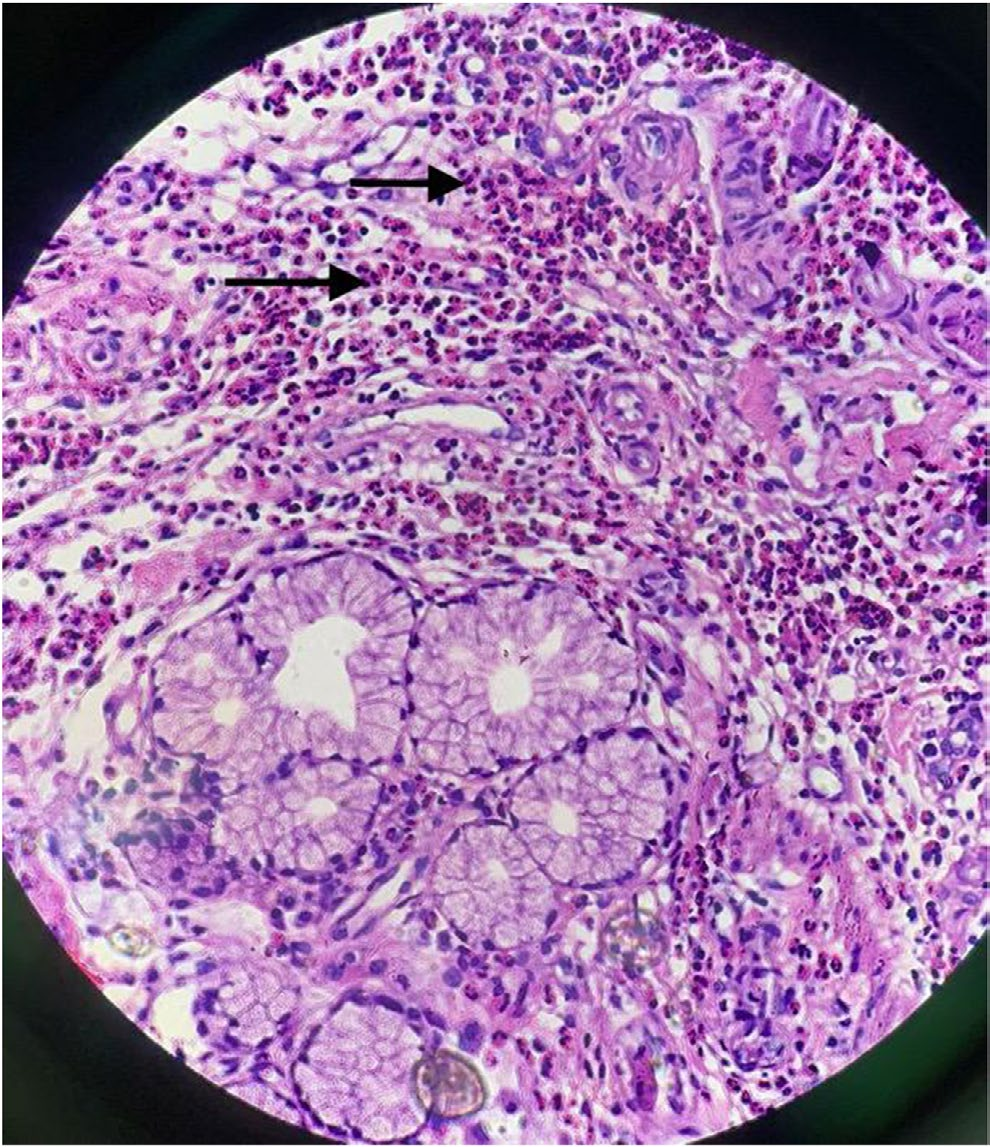
Histopathology from duodenal tissue (40× magnification) showing lamina propria being infiltrated by numerous chronic inflammatory cells, including a fair number of eosinophils.

Esophagogastroduodenoscopy was arranged. During the procedure, the mucosa and other components appeared to be normal. However, with a high index of suspicion of EGE due to a high peripheral eosinophil count, biopsy was taken from multiple sites of esophagus, stomach, and duodenum. Histopathology of esophagus was normal. However, histopathology of stomach and duodenem revealed that both gastric and duodenal lamina propria were infiltrated by numerous chronic inflammatory cells, including a fair number of eosinophils (> 50/HPF) (Figures 1 and 2).

Intestinal parasitosis is quite prevalent in Bangladesh, and our patient presenting with peripheral eosinophilia underwent a stool microscopic examination, which yielded normal results [5]. ELISA for echinococcal antibody was also negative. In order to exclude involvement of other organs, the patient also underwent several tests including liver function, renal function, pulmonary function, echocardiogram (ECG), and troponin I. Despite the medical recommendation for a bone marrow aspiration to further investigate the condition, the patient declined to undergo another invasive procedure following the upper GI endoscopy. Additionally, a colonoscopy was advised to assess potential involvement of the colon, but the patient also refused to proceed with this suggested examination.

Based on these findings, we made a diagnosis of eosinophilic gastroenteritis and started the patient on oral prednisolone 40 mg/day for 2 weeks, with rapid taper over 2 weeks. Patient responded dramatically.

## 4 Follow Up

Within a week, symptoms subsided, the WBC count came down to normal, and the eosinophil count came down to 1%. Treatment was continued, and she was discharged after proper counseling regarding the nature of the disease and the possibility of relapse. After 3 months, she returned for a follow‐up appointment. During this period, she experienced no abdominal pain. Additionally, her eosinophil count and IgE levels remained normal. Details of the investigations are given in Table 1.

**TABLE 1:** Investigations during admission, follow up after 7 days, and follow up after 3 months.

Investigations	Admission	Follow up after 7 days	Follow up after 3 months	Reference value
CBC				
Hb (g/dL)	14.7	13.7	14	12–16
ESR (mm Hr)	25	7	8	0–15
WBC (cells/μL)	28,780	10,800	10,500	4000–11,000
Neutrophil (%)	32	68	68	40–75
Lymphocyte (%)	14	29	30	20–50
Eosinophil (%)	52	1	1	1–6
CRP (mg/L)	115	5	5	< 10
S. creatinine (μmol/L)	70	70	76	55–97
S. bilirubin (μmol/L)	17		17	5.1–20.5
SGPT (U/L)	30		35	10–49
Serum IgE (kU/L)	172		10	0–140 U/L
Stool for ova and parasite	Negative			
ELISA for echinococcus antibody	Negative			

Abbreviations: CBC, complete blood count; CRP, C reactive protein; Hb, hemoglobin; WBC, white blood cell.

## 5 Discussion

Eosinophils are part of the GI mucosal immune system. They are principally associated with parasitic infections and allergic conditions. The pathophysiology of EGE is not well determined. The process starts with recruitment and activation of a large number of eosinophils in the GIT with consequent release of cytokines, giving rise to the wide spectrum of problems [6].

The presentation of EGE depends on the layers involved. Mucosal involvement, which is the commonest form, usually presents with abdominal pain, diarrhea, vomiting, protein losing enteropathy, etc. If the muscle layer is involved, presentation is often obstruction, even perforation. Serosal involvement presents with ascites. Involvement of all three layers has also been reported in rare cases [2]. In our case, based on the symptoms and eosinophilic infiltration of the lamina propria, it was of the mucosal variety.

Due to its non‐specific presentation, it is often difficult to diagnose EGE, especially at an early stage. However, with peripheral eosinophilia present in over two‐third of the cases, a possibility of EGE should be kept in mind when dealing with a patient with bizarre GI symptoms [7]. In our case, due to the severity of abdominal pain, the local hospital clinically suspected acute pancreatitis. However, with normal enzyme levels, no definitive diagnosis was reached. Initial symptom relief was achieved with painkillers, leading to her discharge. This led to a delay in diagnosis and undue suffering for the patient. This is a difficult issue. There have been several reported cases where EGE have both caused and mimicked acute pancreatitis [8, 9, 10, 11, 12]. There has been one where it has mimicked acute cholecystitis, leading to emergency cholecystectomy [13]. This potential for varied presentation and frequent misdiagnosis has led to EGE being referred to the great chameleon.

45% to 60% of EGE patients has a history of allergies, like asthma, allergic rhinitis, atopy, etc. [14]. Our patient did not give any such history, although her daughter was an asthma patient. In rare cases, late cow's milk allergy and even egg allergy has been reported, where avoidance of milk and dairy products led to significant improvement in symptoms [9, 15].

In order to exclude other organ involvement, various tests were conducted, including liver function tests, renal function tests, pulmonary function tests, and cardiac enzyme assessments. Intestinal parasitosis was also excluded, owing to its high prevalence in Bangladesh [5]. However, it is essential to note that the bone marrow test, which is crucial for ruling out hypereosinophilic syndrome, was not performed due to the patient's refusal. Neither was colonoscopy. We recognize these as limitations.

Although an established treatment protocol does not exist, steroid is widely accepted as the mainstay of treatment. Other treatment options include leukotriene inhibitors, mast cell stabilizers, antihistamines, and biological agents [16]. Our patient responded dramatically to oral prednisolone and did not have any relapse in our short follow‐up period.

## 6 Conclusion

EGE is a rare disorder with a non‐specific presentation. This often leads to diagnostic dilemma, misdiagnosis, and delayed diagnosis, resulting in significant suffering for the patient. Diagnosis mainly depends on endoscopic biopsy and histopathology. The treatment approach, with steroid therapy as the widely accepted mainstay, is effective and generally leads to positive outcomes. If a higher index of suspicion is kept in atypical GI presentations, more cases will be diagnosed earlier in the future.

## Author Contributions

**Sumona Islam:** conceptualization, investigation, methodology, resources, writing – original draft, writing – review and editing. **Dewan Saifuddin Ahmed:** conceptualization, investigation, supervision, writing – review and editing. **Nabila Tasneem Khan:** data curation, methodology, software, visualization, writing – review and editing. **Farjana Sultana Rakhi:** project administration, validation, visualization, writing – review and editing.

## Consent

Informed written consent was taken from the patient.

## Conflicts of Interest

The authors declare no conflicts of interest.

## Data Availability

All the data related to this case report are available with the corresponding author and will be available upon request.
